# *Trans* enantiomeric separation of MESA and MOXA, two environmentally important metabolites of the herbicide, metolachlor

**DOI:** 10.1016/j.mex.2022.101884

**Published:** 2022-10-19

**Authors:** Marla R. Bianca, Clifford P. Rice, Robert Lupitskyy, Rebecca E. Plummer, Gregory W. McCarty, Cathleen J. Hapeman

**Affiliations:** a USDA-ARS, Sustainable Agricultural Systems Laboratory, Maryland 20705, USA 22; b TIC Gums, 10552 Philadelphia Rd., White Marsh, MD 21162, USA; c Formerly USDA-ARS, Hydrology and Remote Sensing Laboratory, Maryland 20705, USA; d USDA-ARS, Hydrology and Remote Sensing Laboratory, Maryland 20705, USA

**Keywords:** Chiral Separation, Atropisomers, Enantiomers, UPLC, Mass spectrometry

## Abstract

Complete separation of the *trans*-enantiomers of the two most abundant, persistent polar metabolites of metolachlor, metolachlor ethane sulfonic acid (MESA) and metolachlor oxanilic acid (MOXA), was achieved using UPLC equipped with a reverse phase chiral column and trace detection with an electrospray triple quadrupole mass spectrometer. Various conditions that influenced the separation and instrumental signal were investigated to achieve the optimum separation and instrument response within an analysis time of less than 30 minutes. Different eluting solvent compositions for each metabolite were required for optimized separation of of the 4 enantiomers. Standard curves were responsive to less than 13 ng/mL and 8 ng/mL for the least plentiful MOXA and MESA enantiomers, respectively with a linear coefficient of determination greater than 0.998. Suitability of the method for quantification of the 4 mixed enantiomers of each was demonstrated using natural surface water samples collected from the Choptank River watershed in Eastern Maryland.

• LC chiral separation parameters were varied to achieve optimal separation of the major enantiomers of the two metolachlor metabolites.
• LC/MS-MS parameters were adjusted to maximize response and minimize analysis time.
• Finished methods were used to quantitate enantiomers in archived stream water extracts from agricultural watersheds with corn/soybean production.

## Graphical abstract

**Figure:**
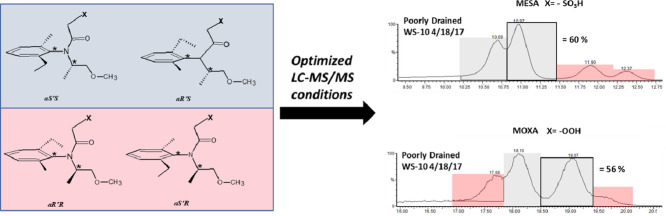

Specifications Table

**Table:** 

Subject area:	
More specific subject area:	Analytical Chemistry
Name of your method:	Chiral separation of enantiomeric herbicide metabolites
Name and reference of original method:	Rice et al. Sci Total Environ 2016 Vol. 560-561 Pages 36-43 and Plummer et al. J Agric Food Chem 2020 Vol. 68 Issue 8 Pages 2297-2305
Resource availability:	*N.A.*

## Method details

### Background

**Figure 1:**
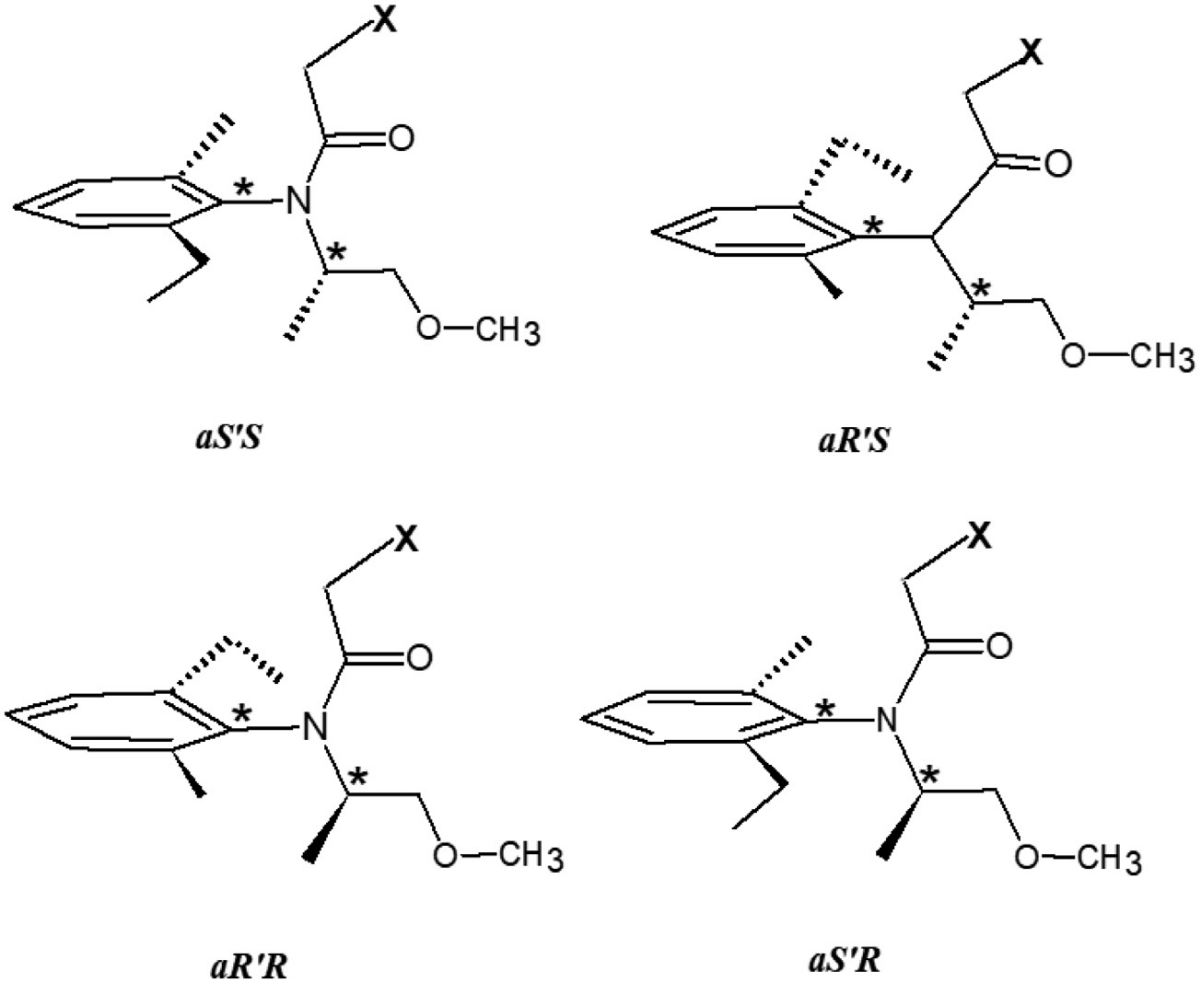
Metolachlor (X = Cl), MESA (X = SO_3_H), and MOXA (X = OOH) consist of two sets of enantiomers due to an asymmetric carbon and the hindered rotation about the phenyl–nitrogen bond.

Metolachlor [2-chloro-*N*-(2-ethyl-6-methylphenyl)-*N*-(1-methoxypropan-2-yl)acetamide] is a common pre-emergent herbicide widely used in agricultural crop production (primarily corn, soybeans and sorghum) [7], [9], and on lawns and turf [21], and is quickly metabolized in soil to two abundant polar metabolites, 2-[2-ethyl-*N*-(1-methoxypropan-2-yl)-6-methylanilino]-2-oxoethanesulfonic acid (MESA) and (2-[2-ethyl-N-(1-methoxypropan-2-yl)-6-methylanilino]-2-oxoacetic acid (MOXA) [1], [2]. Introduced in the US in 1976, metolachlor was a racemic mixture consisting of two sets of enantiomers, 1) the solitary asymmetric carbon (C1) and 2) a hindered rotational axis involving the asymmetrically substituted phenyl group and the partial double bond character of the amide bond (Figure 1) [1], [9]. These two chiral features produce 2 sets of 4 enantiomers of each metabolite, and one would expect equal quantities of each metabolite enantiomer, however, the carbonyl group hinders the *cis* forms with respect to the phenyl ring, resulting in a greater abundance of the 4 *trans* isomers.

The herbicidal activity of metolachlor has long been known to be a function of its chirality [12], specifically, the *S*-configuration has a much larger herbicidal activity [13] than the *R*-configuration. Starting in 1998, the manufacturer introduced a formulation change, from the racemic metolachlor (equal amounts of *S*- and *R*- enantiomers) to the more active *S*-enriched form (88% *S*-metolachlor). By the year 2001, this *S*-enriched formulation was the dominant product applied across the U.S. [20].

### Chemicals and reagents

Standard reference materials for MESA: racemic MESA (Syngenta ID CGA-354743) and separate *S*- (SYN-502271) and *R-* (CGA-380168) enantiomers were obtained from Syngenta (Greensboro, NC). The internal standard, ^13^C_6_-ring-labeled racemic MESA, was obtained from Cambridge Isotopes, (Tewksbury, MA). Standard reference material for racemic MOXA (CGA-51202) and *S*-MOXA (CGA -351916) were secured from U.S. EPA National Pesticide Standard Repository (Ft. Meade). High-performance liquid chromatography (HPLC) grade methanol (MeOH), acetonitrile (ACN), and glacial acetic acid were obtained from Fisher Scientific (Waltham, MA) and 18 MΩ ultrapure organic free water was provided by a Picotech UV Plus system (Hydro Service and Supplies, Gaithersburg, MD). ACS reagent ammonium acetate was obtained from Sigma-Aldrich (St. Louis, MO).

### Field-collected Sample extracts for method testing

Representative sample extracts prepared using an enhanced method [10] were selected from a large archive of extracts spanning more than 15 years from a long-term study of 15 subwatersheds in the Choptank Watershed located in the Delmarva region of MD and reported elsewhere [14], [15], [16]. Four selected samples from each of three years (2010, 2013, 2017) were chosen. Briefly, water samples (1 L) were collected and filtered (0.7 μm GF/F) and then fractionated using SPE cartridges (Oasis HLB, 500 mg) allowing recovery of MESA and MOXA from elution with methanol (12 mL) and acetonitrile (6 mL) after concentration with a gentle nitrogen stream to 5 or 10 mL. These concentrated volumes were adjusted to assure that integrated analyte peaks remained within the respective limits of detection discussed below.

### Instrument Setup

The enantiomeric separations were achieved using an Acquity H-Class Plus UPLC (Waters Corporation, Milford, MA) equipped with a reverse phase chiral column Daicel Chiralpak QN-AX (150 mm × 4.6 mm, 5 μm, Chiral Technologies, Inc., West Chester, PA) with a QN-AX guard column (4 mm × 10 mm, 5 μm). Analyses were performed using a quadrupole mass spectrometer, Xevo TQS micro step wave MS/MS (Waters Corporation), operated in electrospray ionization (ESI) using both positive and negative modes in multiple reaction monitoring mode (MRM). Cone gas and desolvation gas were nitrogen supplied by a Genius XE 35 nitrogen generator (Peak Scientific, Inchinnan Scotland) and the collision gas is argon (Airgas USA, Independence, OH). The MS/MS tuning parameters and peak detections were optimized using Intellistart (program within MassLynx) (Waters Corporation). The transition ions for qualitative screening used three mass pairs each for MESA (pos. ESI), and MOXA (neg. ESI) (Table 1).

**Table 1:** MRM Transitions for MOXA, MESA, and ^13^C-MESA in ES+ and ES- Ion Modes

compound	ion mode	parent *(m/z)*	daughter *(m/z)*	cone (V)	collision (eV)
MOXA	ESI -	278.14	206.16*	30	10
MESA	ESI +	330.11	298.12*	42	14
^13^C-MESA	ESI -	334.10	141.03*	74	26
^13^C-MESA	ESI +	336.16	304.17*	38	16

⁎ Quantification ion

### Optimization for separations and quantitation of 4 isomers of MESA and MOXA

Optimizing parameters included changing column temperatures, mobile phase solvent ratios, buffer concentrations and pH, and running solvent flow rates. Various combinations were adjusted to achieve optimal separation while maintaining adequate sensitivity which also involved adjustments of cone gas, desolvation gas flows and temperature, and injection volumes. The parameters that most influenced the separation and instrument response were mobile phase solvent composition, pH of the ammonium acetate buffer, column temperature, and desolvation temperature. Previous separations of MESA produced 3 peaks using 90:10, methanol: 50 mM ammonium acetate buffer pH 5 [8], [16], [17]. However, adding acetonitrile and mixing different ratios of these solvents, allowed for better separation of the enantiomers of each metabolite.

Optimized isocratic separations of MESA versus MOXA were found to have very different solvent ratio compositions (acetonitrile, ammonium acetate buffer, water, and methanol). However, for both compounds, methanol increased the instrument response but not the separation; water decreased the instrument response and increased the separation slightly and the retention time; acetonitrile decreased the instrument response but increased the separation. Additionally, modifying the ammonium acetate buffer to pH 4.7 overall decreased the sensitivity for both metabolites but helped with separation. For MESA, this modification allowed for greater separation of the *S*-peaks and did not significantly decrease the signal while still maintaining a method run time of less than 30 minutes. However, for MOXA, a pH decrease from 5 to 4.7 slightly improved separation, but reduced sensitivity and increased retention times even more. Therefore, we used the original buffer of pH 5 for MOXA because a pH 5 buffer still allowed for all 4 isomers to be separated.

Column temperatures were varied from 25 to 15°C (in intervals of 5°C). A decrease in column temperature to 15°C increased the separation for both MESA and MOXA; therefore, 15°C was considered the optimal column temperature. To achieve sample runs of less than 30 min., a flow rate at 0.6 mL/min was used. Higher flow rates were prohibited as they exceeded the pressure limit of the column and decreased separation. Desolvation temperatures of 450, 500, and 600°C were tested, and 600°C produced the best results.

Taking all the factors into consideration, the optimal chromatographic conditions for separation of the 4 *trans*-isomers of MESA was a column temperature of 15°C, flow rate of 0.6 mL/min an isocratic solvent system of 48:1:51- methanol: 50 mM ammonium acetate (pH 4.7): 2% 18 MΩ ultrapure organic free water in acetonitrile; for MOXA the same column temperature and flow was used with the isocratic solution of 81:8:11- acetonitrile: 50 mM ammonium acetate (pH 5) and18 MΩ ultrapure organic free water. The optimal instrument response for both MESA and MOXA was observed using cone gas at 100 L/h, desolvation gas at 1000 L/h, desolvation temperature of 600°C, and injection volume of 5 µL for MESA and 10 µL for MOXA (the higher injection volume helps overcome the lower sensitivity found for MOXA which were caused by the greater amount of acetonitrile and water used in this mobile phase as both have a tendency to suppress ESI response for this instrument [4].

**Figure 2:**
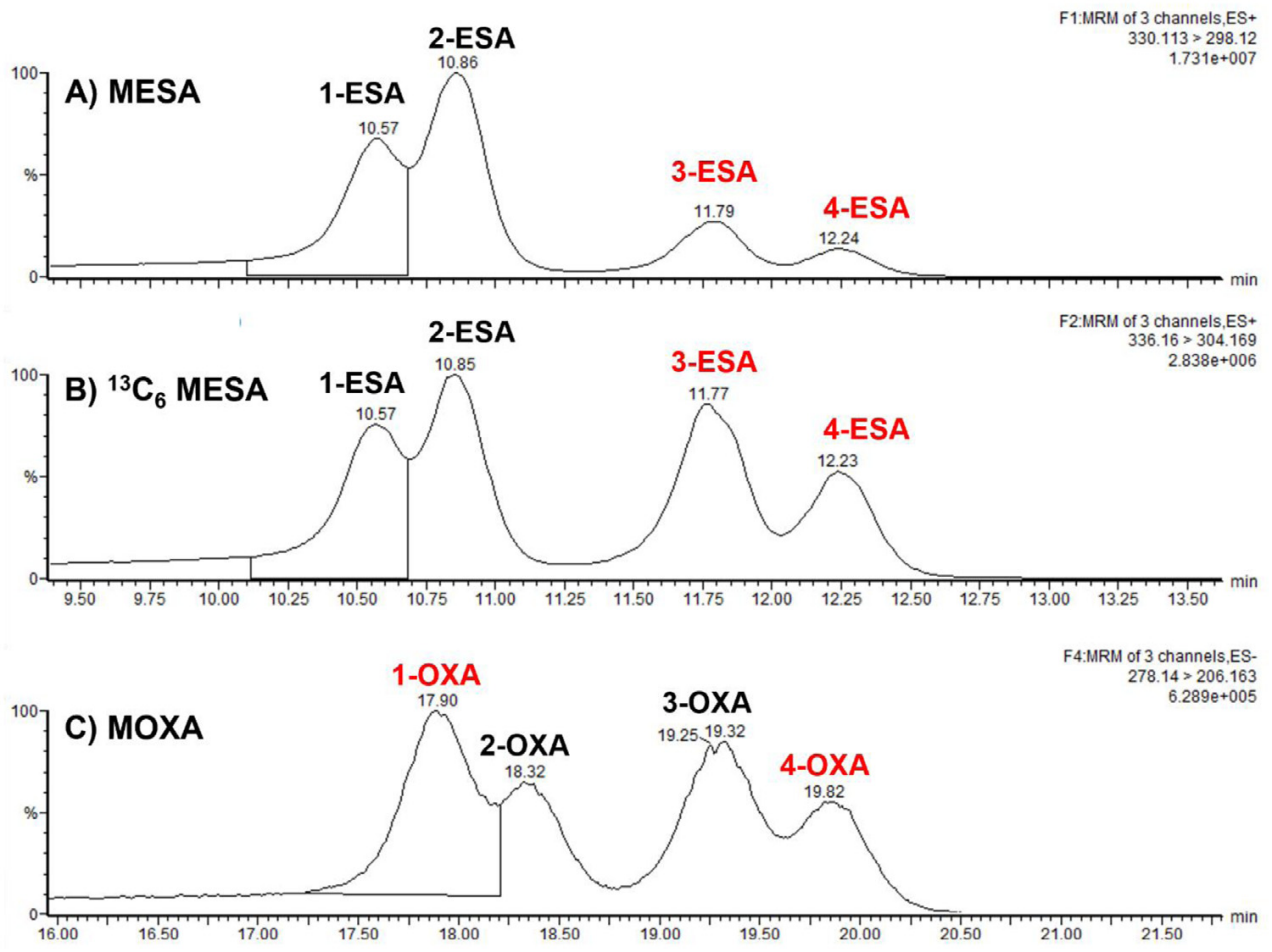
LC MS/MS chromatogram of Top: MESA (75% *S* and 25% *R*), Middle: ^13^C_6_-MESA racemic), and Bottom: MOXA (racemic). Peaks labels by order of elution 1 to 4- ESA and 1 to 4- XA, for MESA and MOXA peaks respectively. Black labels refer to the *S* and red labels refer to *R* enantiomers.

Using these optimized parameters and for the purposes of this study, the 4 peaks (for each metabolite) were labeled by their elution order: 1- to 4- ESA and 1- to 4-OXA for MESA and MOXA, respectively (Figure 2). The elution order of the *S* and *R* enantiomers are not the same for MESA and MOXA; the *S*-enantiomer peaks for MESA are the first two (1-ESA and 2-ESA) and the *R* peaks are the last two (3-ESA and 4-ESA), while for MOXA, the *S*-enantiomer peaks (2-OXA and 3-OXA) elute between the *R* peaks (1-OXA and 4-OXA). *S-* versus *R-*enantiomer peak elution was confirmed by comparison of retention time to *S-*enantiomer only MESA and *S*-enantiomer only MOXA standards).

### *Calibration and Sensitivity (LOD and LOQ)*

Individual 6-point calibration curves were developed for both MESA and MOXA enantiomers. The preparation of the calibration curves for each of the MESA enantiomers was straight forward since separate standards for the *S* and *R* enantiomers were available. This also facilitated the separation method development as we could adjust the *S* and *R* concentrations to reflect the reduced amount of the *R* enantiomers that are currently observed in field-collected stream waters. Thus, we prepared standards which had 3 times more of the *S*-enantiomer than the *R*-enantiomer. To complete the calibration curve for MESA it was also necessary to adjust each of the rotamer forms for each rotamer in each enantiomer to reflect the fact that they occur naturally in the standards as different peak area proportions (Figure 2. Top: MESA chromatogram). This resulted in the following 6-point calibrations curve ranges for *S* rotamer pair in MESA, 1-ESA, 27 to 864 ng/mL and for 2-ESA, 48 to 1560 ng/mL and for the R rotamer pair in MESA, 3-ESA, 18 to 564 ng/mL and 4-ESA, 3 to 243 ng/mL. An internal standard was also used for all the analyses of all samples using ^13^C_6_-ring-labeled racemic MESA (25 µL of a 20 mg/L solution per 1 mL) yielding 500 ng/mL (total isomer concentration) per injection vial. MESA enantiomers were all matched with the ^13^C_6_-ring-labeled racemic MESA peaks and quantitated using compound and the isotope internal standard method (MassLynx 4.3- Waters Corporation).

The calibration of the MOXA enantiomers was more challenging because pure *S*- or *R*-MOXA was not available. Therefore, for MOXA, a racemic standard was employed varying from 35 ng/mL to 2000 ng/mL MOXA (total enantiomeric concentration). For MOXA isomer quantitation, it was necessary to convert this total concentration to the quantities for each individual enantiomer. Because the standard has equal *S* and *R* enantiomers, the total values were divided in half to produce equal concentration for the rotamer pairs. Next the final concentrations for each isomer in these pairs were adjusted to reflect the relative areas counts for each rotamer peak as shown in Figure 2 (Bottom panel separated MOXA). Careful repeat analyses of 10 racemic MOXA standards determined that the proportion of the rotamer pair was approximately 3 parts the larger peak (1-OXA, *R*) and 2 parts for (4-OXA, *R*) and, likewise, this proportion held for the larger (3-OXA, *S*) versus the smaller (2-OXA, *S*) peak, which was similar to the 3:2 ratio for the larger peak observed for MESA rotamers. Thus, for the 2000 ng/mL MOXA, the final isomer values for the R enantiomers were 640 ng/mL for 1-OXA and 360 ng/mL for 4-OXA and for the *S* enantiomers they were 320 ng/mL for 2-OXA and 680 ng/mL for 3-OXA. Therefore, the ranges for 6-point standards were 11 to 640 ng/mL for 1-OXA (largest *R* peak), 12 to 680 ng/mL for 3-OXA (largest *S* peak), 6 to 320 ng/mL for 4-OXA (smallest *R* peak), and 6 to 360 ng/mL for 2-OXA (smallest *S* peak). Making these adjustments allowed individual isomer quantitation to be performed using the racemic MOXA standard. The retention times for the MOXA enantiomers were based on the relative retention times to the labelled internal standard (^13^C_6_-ring-labeled racemic MESA), and each was quantitated by the external standard method.

**Figure 3:**
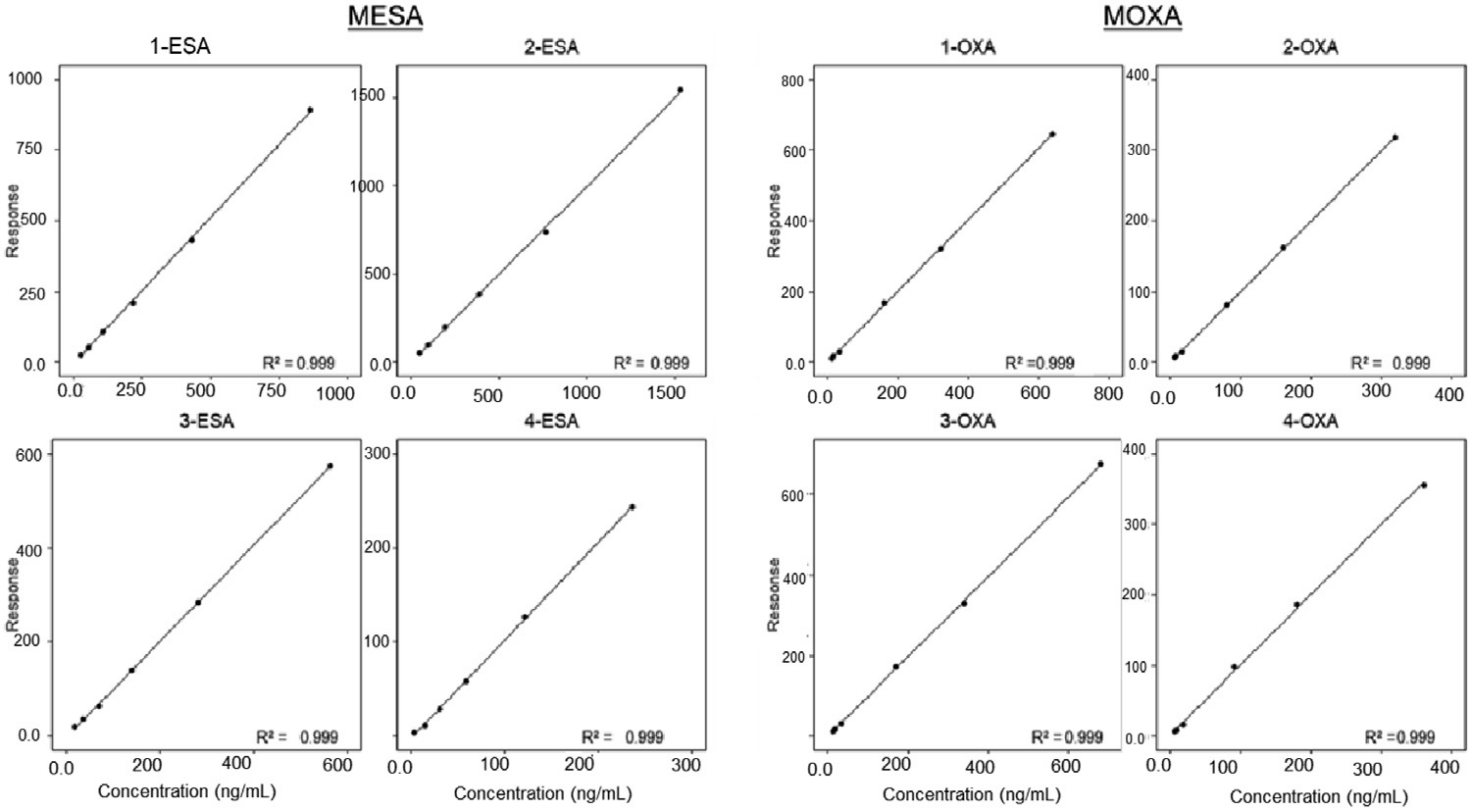
Example set of calibration curves for *trans*-enantiomers of MESA using the internal standard method (left) and of MOXA using the external standard method (right).

Calibration curves for both MESA and MOXA had high coefficients of determination for all 4 of their isomers (R^2^>0.998). Examples of the calibration curves for the 4 MESA and 4 MOXA enantiomers are shown in Figure 3.

The calibration intercept approach was used to determine the limit of detection (LOD) and limit of quantification (LOQ) for each isomer of both MESA and MOXA and using ESI positive for MESA and ESI negative for MOXA [3]. The LOD was calculated by multiplying three times the standard error of the intercept of the calibration curve, then dividing by the slope; the LOQ was calculated similarly by multiplying by ten (instead of three). For MESA using ESI positive: 1-ESA, 2-ESA, 3-ESA, and 4-ESA the LOD's were 7, 16, 6, 4 ng/mL respectively, and the LOQ's were 22, 53, 19, 13 ng/mL, respectively. The LOD for each of the isomers were below that of the lowest MESA standard and LOQ for each isomer were below that of the lowest MESA standard, except for 2-ESA (lowest standard was 48 ng/mL; another 6-point calibration curve was run on a different day and had a LOQ of 42 ng/mL which was below the concentration of the lowest standard for 2-ESA). Additionally, the lowest standard (75 ng/mL total MESA) was run 10 times to evaluate the accuracy as well as precision of this low concentration. Each of the measured isomers were approximately 1% or less different than their respective expected concentration and had a standard deviation of < 1.1 ng/mL (Table 2).

**Table 2:** Replicate measurements of the lowest MESA standard

MESA	Enantiomer	standard conc (ng/mL)	measured conc (n=10) (ng/mL)	% diff
100 ng/L	1-ESA	27	27.2 (0.7)	0.7
	2-ESA	48	49.2 (1.1)	1.1
	3-ESA	18	18.2 (0.4)	0.4
	4-ESA	7	7.2 (0.2)	0.2

For MOXA using ESI negative, the LOD's for 1-OXA, 2-OXA, 3-OXA, and 4-OXA were 3, 2, 5, 5 ng/mL, respectively, and the LOQ's were 12, 8, 19, 17 ng/mL, respectively. The LOD for each of the enantiomers were below that of the lowest MOXA standard, however the LOQ for each isomer was not below that of the lowest MOXA standard (for reference a sample with a total MOXA concentration of 100 ng/L would be above the LOQ).

### Applicability of Method

**Figure 4:**
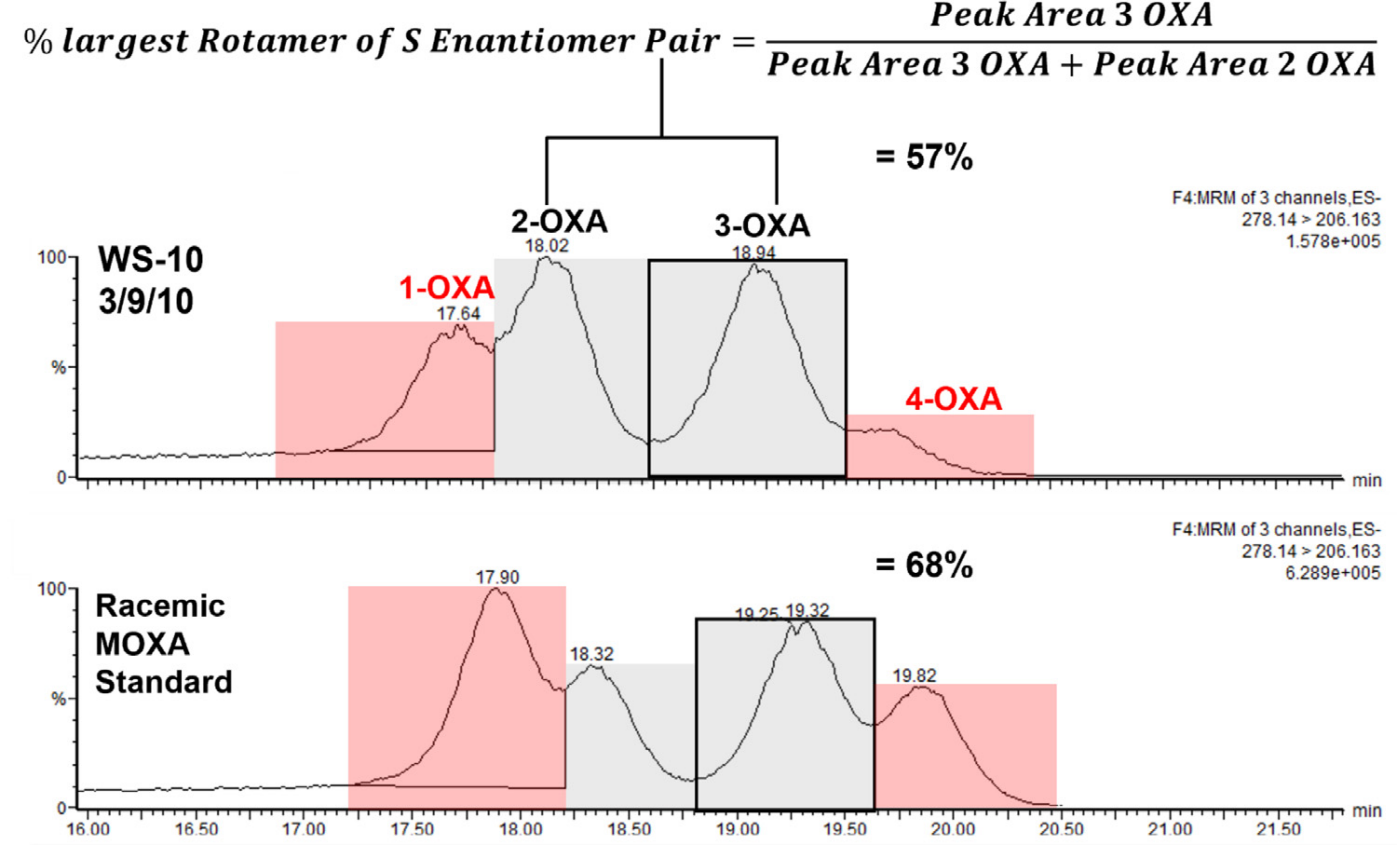
Demonstration of shifts of rotamers occurring in field samples, i.e peak ratio in MOXA standard versus a 2010 extraction of MOXA from subwatershed 10 in our Choptank collections. Example of procedure to determine rotamer peak percent of enantiomer pairs.

The applicability of the developed methods for MESA and MOXA separation and quantification was tested using samples collected and extracted from sites in the Choptank River watershed in Eastern Maryland (4 sites from three different years: 2010, 2013, and 2017). The specific feature examined in these samples was the different rotamer ratios for each of the enantiomers of the *S* and *R* forms. To calculate these values, the percentage of the largest rotamer peak for each pair was calculated by dividing the largest peak area for the pair by the total area of the sum of the peak areas. An example of this method is demonstrated in Figure 4. In this figure a MOXA chromatogram from a field sample (WS-10 from 3/9/10) was compared to a typical chromatogram of the racemic MOXA standard. Even compared to the MOXA standard, pattern differences were clearly present, 1) the ratio of the two *S*-enantiomers (2-OXA and 3-OXA) are very different from those in the MOXA standard; and 2) the two *R*-rotamer peak areas (1-OXA and 4-OXA) in the field sample are reduced compared to these two rotamer peaks for the *R*-enantiomer in the standard. This reduction in the two *R*-enantiomer peaks was expected since this metabolite was likely mixed with products formed from *S*-enriched metolachlor which was introduced into field application in 1998 and compared to a MOXA standard matching the racemic composition of racemic metolachlor that it replaced.

Using our 4-peak method on the selected Choptank subwatershed samples we divided those samples into two sets, one set from the well-drained section (WDU) of the watershed and the other set from a poorly drained section (PDU) [6]. The individual results for the *S*-enantiomer peaks for MESA and MOXA are shown, Table 3. Recoveries for total MESA and MOXA have been reported previously [11] as 91±13-MESA; 87 ± 15-MOXA (total concetrations). For this study, each LOD value listed in the “Calibration and Sensitivity section”, i.e. 7, 16, 6, 4 ng/mL MESA and 3, 2, 5, 5 ng/mL MOXA, was compared to the values in Table 3 and all reported values in Table 3 were greater than these detection limits.

**Table 3:** Rotamer peak ratios and concentration results for 4 *trans* enantiomers of MESA and MOXA based on the 4-peak method for three separate years, 2010, 2013 and 2017 in 1 mL extracts from water samples from 4 sample locations, 2 located in a well drained area and 2 collected in a poorly drained area.

	MESA						MOXA					
Sample Names	*S*-Enantiomer			*R*-Enantiomer			*S*-Enantiomer			*R*-Enantiomer		
Well Drained Area (WDU)	1-ESA	2-ESA	% 2-ESA for Pair	3-ESA	4-ESA	% 3-ESA for Pair	2-OXA	3-OXA	%3-OXA of Pair	1-OXA	4-OXA	%1-OXA of Pair
	ng/mL	ng/mL	Percent	ng/mL	ng/mL	Percent	ng/mL	ng/mL	Percent	ng/mL	ng/mL	Percent
WS2 3/9/10	220	310	57.9	190	82	69.3	70	120	62.6	69	28	71.1
WS2 1/22/13	120	170	58.3	79	35	69.3	60	110	63.3	50	20	71.4
WS2 4/18/17	130	190	59.6	40	38	68.9	60	100	60.5	41	17	70.7
WS6 3/9/10	240	340	58.6	240	110	69.4	80	110	60.0	84	31	73.0
WS6 1/22/13	130	180	59.2	120	52	69.7	50	80	59.7	60	24	71.4
WS6 4/18/17	130	200	61.1	120	52	69.5	60	90	60.3	60	22	73.2
		Average	59.1%		Average	69.4%		Average	60.0%		Average	72.6%
Poorly Drained Area (PDU)	1-ESA	2-ESA	% 2-ESA for Pair	3-ESA	4-ESA	% 3-ESA for Pair	1-OXA	2-OXA	% 2-OXA for Pair	3-OXA	4-OXA	% 3-OXA for Pair
	ng/mL	ng/mL	Percent	ng/mL	ng/mL	Percent	ng/mL	ng/mL	Percent	ng/mL	ng/mL	Percent
WS10 3/9/10	150	230	60.4	120	57	67.0	70	100	56.9	42	16	72.4
WS10 1/22/13	90	150	62.0	63	27	70.4	60	80	57.0	27	11	71.1
WS10 4/18/17	90	140	60.0	51	23	68.9	50	60	55.9	24	8	75.0
WS13 3/9/10	140	200	58.9	140	59	70.5	30	40	56.9	28	10	73.7
WS13 1/22/13	90	140	59.8	78	36	68.6	30	40	57.7	25	9	73.5
WS13 4/18/17	100	150	60.5	79	32	71.1	30	40	55.9	22	10	68.8
		Average	60.3%		Average	69.4%		Average	56.7%		Average	72.4%
WDU vs. PDU	t-test of averages	Not Significant			Not Significant	t-test of averages	Significant			Not Significant		

Comparing the two drainage locations, for most of the enantiomers there is little change in rotamer composition with drainage, with the exception of the *S* enantiomers of MOXA, where there appears to be a consistently lower percent of the two *S*-enantiomers in the PDU samples. Testing the differences using a student t-test of the means indicated a significant difference (p<0.001) for the MOXA *S*-rotamer pairs between the samples in the well-drained upland location versus the poorly drained upland location. Thus, it seems that some environmental process had a major influence on the rotamer composition of *S*-MOXA rotamer pair. These findings demonstrate the utility of gaining information on the 4-isomers of these two significant metabolites of metolachlor and how they offer a means of examining divergence of environmental fate and transport of MESA and MOXA. Showing these differences with so few data, implies that more studies using a larger dataset should uncover more differences that may reveal information about environmental processes of the isomers of these compounds. Such divergence may reflect several factors involved in their origins from a common source, metolachlor, and the various processes involved in their transport through the soil and passage with groundwater to the receiving streams and fate in agricultural watersheds. While the results in Table 3 focus mostly on the ratio of the rotamer peaks, data are also provided for the concentrations expressed as amount in each 1 mL injection. To convert these concentrated 1 mL extract concentration values to whole 1-L sample concentration, it requires multiplying them by the dilution factors (5 or 10x) that were used to keep the integrated peaks within the linear range of the standards. These resulting thousands of nanogram values are the total gram amounts in each 1-L sample, and these are typically converted to microgram amounts. Therefore, μg/L is the usual unit for reporting water sample concentration for these compounds.

### Additional information

#### Rationale for developing improved separation method

MESA and MOXA are exceptionally stable moving through groundwater with minimal degradation and are typically detected at larger concentrations than metolachlor in ground and surface waters in watersheds with metolachlor usage [18]. They also possess key chemical properties that are very similar to nitrate and can serve as conservative transport analogs for nitrate from cropland [10]. Furthermore, during the glutathione-mediated degradation pathway of metolachlor, the parent stereochemistry is retained in the formation of both MESA and MOXA [9], [17], [19]. Thus, the temporal change in chirality near the turn of the century can be used to determine groundwater residence times [17].

MESA and MOXA generally occur together in natural waters collected from watersheds with metolachlor usage and can be excellent tools for studying biogeochemical and transport processes in cropland-impacted waters. MESA and MOXA are unique environmental tracers in that they are both highly soluble metabolites of metolachlor applied to croplands, but they may have divergent watershed fates. Study of the correlations of MESA and MOXA concentration and enantiomeric composition in ground and surface waters can provide insights on transport and fate mechanisms within agricultural watersheds.

Environmental monitoring of MESA and MOXA has previously been performed without enantiomeric separation [5], [11], [22]. Chiral separation for three of the 4 major enantiomeric peaks of MESA was first reported by Kabler [8]. In other work, we have modified the Kabler method and applied it to surface water samples for MESA analyses [16], [17]. Separation, using reverse phase LC-ESI-MS/MS, of the 4 *trans*-isomers of MESA has been elusive and a method for separating isomers of MOXA has yet to be reported. In this study we report separation of all 4 *trans*-isomers of MESA using reverse phase LC-ESI-MS/MS and extended the methodology for individual enantiomeric separation of MOXA. Optimal separation of all *trans*-isomers of MESA and MOXA was achieved using a QN-AX chiral column (Chiral Technologies, Daicel Group, West Chester, PA).

Ethics statements

## CRediT authorship contribution statement

**Marla R. Bianca:** Investigation, Methodology, Conceptualization, Validation, Formal analysis, Writing – original draft. **Clifford P. Rice:** Conceptualization, Methodology, Resources, Writing – review & editing, Supervision. **Robert Lupitskyy:** Investigation, Writing – review & editing. **Rebecca E. Plummer:** Investigation, Writing – review & editing. **Gregory W. McCarty:** Writing – review & editing. **Cathleen J. Hapeman:** Writing – review & editing.

## Declaration of Competing Interest

The authors declare that they have no known competing financial interests or personal relationships that could have appeared to influence the work reported in this paper.
